# Impact of the first COVID-19 lockdown in Germany on the rate of acute infections during intensive chemotherapy for Hodgkin lymphoma

**DOI:** 10.1007/s15010-022-01765-3

**Published:** 2022-02-19

**Authors:** Anne Sophie Jacob, Helen Kaul, Michael Fuchs, Sarah Gillessen, Stefanie Kreissl, Annette Pluetschow, Jesko Momotow, Valdete Schaub, Andreas Huettmann, Mathias Haenel, Andreas Zimmermann, Judith Dierlamm, Julia Meissner, Stephan Mathas, Sonja Martin, Andreas Engert, Michael Hallek, Peter Borchmann, Clara Lehmann

**Affiliations:** 1 Department I of Internal Medicine, Center for Integrated Oncology Aachen Bonn Cologne Duesseldorf, University of Cologne, Faculty of Medicine and University Hospital Cologne, Kerpener Str. 62, 50937 Cologne, Germany; 2 CECAD Center of Excellence on Cellular Stress Responses in Aging-Associated Diseases, Center for Molecular Medicine Cologne, Cologne, Germany; 3 German Hodgkin Study Group (GHSG), University of Cologne, Cologne, Germany; 4 Eberhard-Karls-Universität-Universitätsklinik Tübingen, Inneren Medizin II, Tübingen, Germany; 5 Universitätsklinik Essen, Klinik Für Hämatologie/WTZ Ambulanz, Essen, Germany; 6 Klinikum Chemnitz, Krankenhaus Küchwald, Klinik Für Innere Medizin III/Studiensekretariat, Chemnitz, Germany; 7 Medizinische Klinik Und Poliklinik III, Klinikum Der Universität München, LMU München, Munich, Germany; 8 Abt. Hämatologie/Onkologie, Onkologisches Zentrum, Universitätsklinikum Hamburg-Eppendorf, Hamburg, Germany; 9 Universitätsklinikum Heidelberg, Medizinische Klinik Und Poliklinik V, Heidelberg, Germany; 10 Charite Campus Benjamin Franklin, Universitätsmedizin Berlin, Hämatologie, Onkologie u. Tumorimmunologie, Berlin, Germany; 11 Robert-Bosch-Krankenhaus Stuttgart, Innere Medizin II, Hämatologie/Onkologie, Stuttgart, Germany; 12 Center for Molecular Medicine Cologne (CMMC), 50937 Cologne, Germany; 13 German Center for Infection Research (DZIF), Bonn-Cologne, Cologne, Germany

**Keywords:** COVID-19, Hodgkin lymphoma, Infection, Prophylaxis

## Abstract

**Purpose:**

Evidence on the effect of self-protection via social distancing and wearing face-masks on infections during chemotherapy is currently not available. We asked if the occurrence of acute infections during chemotherapy for advanced-stage Hodgkin lymphoma (HL) decreased when COVID-19 protection measures were in effect.

**Methods:**

We analyzed the occurrence of infections during all documented eBEACOPP cycles starting between 01 March and 30 June of 2017 to 2020 in patients treated within the GHSG HD21 study in Germany and compared the infection rates and characteristics by logistic regression models and means of descriptive statistics.

**Results:**

We analyzed 911 cycles of 313 adult patients treated with 4 to 6 cycles of eBEACOPP. We found a significant decrease in the occurrence of infections during chemotherapy for HL during COVID-19 lockdown from 131 (19.6%) of 670 cycles in 2017–2019 to 30 (12.6%) of 239 cycles during COVID-19 lockdown [OR 0.574 (95% CI 0.354–0.930), *P* = 0.024]. The strongest effect was evident for unspecified infections with 39 cycles (5.8%) during 2017–2019 in comparison to 5 cycles (2.1%) during COVID-19 lockdown. 20 (24.1%) of 83 patients had an infection during the COVID-19 lockdown versus 99 (43.2%) of 229 patients in the years 2017–2019 (*P* = 0.0023).

**Conclusion:**

The significant decrease of infections during chemotherapy for HL during COVID-19 lockdown reveals the protective measures’ potential to shield patients from transmissible pathogens. We conclude that these measures could be recommended for HL patients at risk for infections during chemotherapy.

**Supplementary Information:**

The online version contains supplementary material available at 10.1007/s15010-022-01765-3.

## Introduction

Acute infections and fever under chemotherapy are an important cause of morbidity and mortality in hematologic malignancies [1–8]. For patients with advanced-stage Hodgkin lymphoma (HL) in particular, treatment-related morbidity rates of up to 66% can be observed, depending on the chemotherapy’s intensity [3, 4, 9, 10]: treatment-related Common Terminology Criteria for Adverse Events (CTCAE) Grade 3/4 infections are documented in up to 17% of the patients and febrile neutropenia in 33% of the patients [10]. Therefore, prophylactic anti-infective medication is prescribed frequently, although evidence for positive effects in patients with lymphatic malignancies is limited [5, 8]. Another measure to decrease the rate of infections might be prophylactic self-isolation and avoiding contact to potential sources of infection. In addition, wearing face masks in case of unavoidable contacts might protect against infectious diseases. However, it is still an unanswered question, if these measures are actually effective in this regard. Accordingly, current guidelines do not cover them so far [5, 8].

On January 27, 2020, the first case of an infection with SARS-CoV-2 in Germany was reported. Subsequently, infection rates for SARS-CoV-2 increased rapidly throughout the country so that the German government implemented several protection measures to slow down the spreading of the virus, following recommendations by the World Health Organization (WHO) [11–13]. These measures included closing non-essential businesses, schools, universities and gastronomy, implementing the obligation to wear facemasks in public spaces and most importantly, prophylactic social distancing [11, 14]. Possibly due to the generally high acceptance of these measures, the infection rates continuously dropped over the following months from a maximum of approximately 6500 new infections per day in the beginning of April to around 500 new infections per day in Mid of June 2020 [15]. The protection measures in the public health system apparently had an impact on reducing the rate of infection with SARS-CoV-2 in Germany [16].

This observation raises the question whether these measures would generally prevent the spreading and transmission of respiratory viruses causing influenza and other infections through droplets or direct contact and thereby also reduce the incidence of various infectious complications in cancer patients treated with chemotherapy.

Between July 2016 and August 2020, thus covering periods before and during lockdown measures, we have conducted a controlled, prospective, randomized study in adults with newly diagnosed, advanced-stage Hodgkin lymphoma (GHSG HD21, NCT02661503). Patients in the standard group were treated with eBEACOPP (dose-escalated bleomycin, etoposide, doxorubicin, cyclophosphamide, vincristine, procarbazine and prednisolone), an intensive outpatient polychemotherapy regimen. Fever and infections are the most frequent and clinically relevant complications of this treatment [3]. Consequently, the HD21 trial provides a uniformly treated and well-documented patient cohort, which might allow describing differences between the periods before and during lockdown in the incidence of infections or fever and possibly to derive recommendations from these observations.

We thus aimed to evaluate whether the occurrence of acute infections during chemotherapy for advanced-stage HL decreased since the Corona Virus Disease 2019 (COVID-19) protection measures have been in effect in this well-defined and controlled study population being at high risk for infectious complications.

## Methods

The main aims of our analysis were to assess whether the rate of chemotherapy cycles with acute infections had decreased since COVID-19 protection measures were in effect and to explore possible confounders for a change in infection rates. Our analysis is based on all documented eBEACOPP cycles that started between 01 March and 30 June of any year within the GHSG HD21 study among patients recruited in Germany. Cycles starting between March and June 2020 are being compared with cycles starting between March and June of 2017–2019. This sample was chosen to account for seasonal variation in infection rates. Given that the duration of study treatment is about 3–5 months and hence single patients are not exclusively treated within or outside of the period of interest (01 March–30 June of any year), we analyzed chemotherapy cycles rather than patients and selected those cycles that started in the respective period.

HD21 is an international, randomized phase III study for patients with newly diagnosed advanced stages of HL, including Ann Arbor stages IIB (with a large mediastinal mass or extranodal involvement as risk factors), IIIA/B, and IVA/B. Patients at the age of 18–60 years were randomized to receive PET-guided 4–6 cycles of either eBEACOPP or the experimental BrECADD (brentuximab vedotin, etoposide, cyclophosphamide, doxorubicin, dacarbazine and dexamethasone) regimen (Supplementary material Appendix). The study is performed in accordance with the Declaration of Helsinki and the International Conference on Harmonization guidelines for good clinical practice and registered at ClinicalTrials.gov (NCT02661503). The institutional review board approved this study and all subjects gave written informed consent.

Recruitment for the study started in July 2016 and was completed in August 2020; however, as results are still pending, we limited our analysis to patients in the control group receiving eBEACOPP. Our analyses are based on the database excerpt for the 9th statistical monitoring report with data cut-off on November 02, 2020. Of note, these are preliminary data and queries may be pending. Acute infections were documented either on the chemotherapy eCRF, including CTCAE grade, type of etiologic agent (bacterial, viral, fungal or not done) and whether or not the infection caused febrile neutropenia, or, in case of serious adverse events (SAEs), in the safety database including CTCAE grade and Medical Dictionary for Regulatory Activities (MedDRA) code. All infections, for which no etiologic agent and no specific MedDRA code was available, were classified as “infection not otherwise specified (NOS)”.

Main endpoint was the rate of cycles with acute infections, including any documentation of viral, bacterial, fungal and unspecified infections of CTCAE grade 1–4, febrile neutropenia, and any SAE of a predefined list of MedDRA codes (Supplementary material Appendix) during chemotherapy. Secondary endpoints included infection characteristics and the use of supportive measures such as hospitalization, dose level reductions, and intensified antibiotic prophylaxis. As a sensitivity analysis, we also analyzed the rate of infections within the defined period per patient.

We used means of descriptive statistics to explore primary and secondary outcomes and mixed effects logistic regression models with random intercepts to compare outcomes between cycles starting within the COVID-19 protection period (covered by the period 01 March–30 June 2020) with our reference sample (i.e. cycles starting between 01 March and 30 June of 2017–2019). All regression models were adjusted for age of the patient, cycle number, and white blood cell (WBC) count at day 1 of the respective cycle. We used SAS, version 9.4 (SAS Institute) for statistical analyses.

## Results

**Fig. 1:**
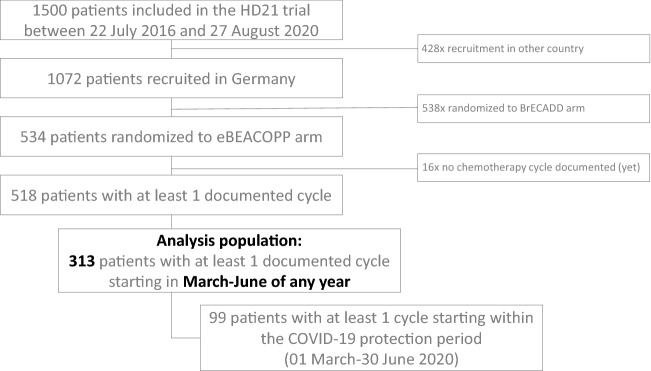
Analysis set. *eBEACOPP* dose-escalated bleomycin, etoposide, doxorubicin, cyclophosphamide, vincristine, procarbazine and prednisolone. *BrECADD* brentuximab vedotin, etoposide, cyclophosphamide, doxorubicin, dacarbazine and dexamethasone

A total of 911 eBEACOPP cycles from 313 patients was analyzed in this study (Fig. 1). The female:male ratio was 46:54 and median age 32 years (range 18–60 years) in the analyzed patient cohort. There were 99 patients with at least 1 cycle starting within the COVID-19 protection period (01 March–30 June 2020). The baseline demographic characteristics are shown in Table 1.

**Table 1:** Baseline characteristics of analyzed and non-analyzed patients

	Not analyzed (*N* = 205)	Analyzed (*N* = 313)	Total (*N* = 518)
Age (years)			
Median (range)	31 (18–60)	32 (18–60)	31 (18–60)
Sex			
Female	89 (43%)	143 (46%)	232 (45%)
Male	116 (57%)	170 (54%)	286 (55%)
Time of recruitment			
Median (range)	10/2018 (07/2016–08/2020)	02/2019 (11/2016–06/2020)	12/2018 (06/2016–08/2020)
Number of documented cycles			
Median (range)	4 (1–6)	4 (1–6)	4 (1–6)
Number of documented cycles in March to June of any year			
Median (range)	–	3 (1–6)	1 (0–6)
Survival status			
Alive	203 (99.0%)	310 (99.0%)	513 (99.0%)
Death (1st-line infection)	1 (0.5%)	–	1 (0.2%)
Death (other)	1 (0.5%)	3 (1.0%)	4 (0.8%)

Data presented as *n* (%), unless otherwise indicated

**Fig. 2:**
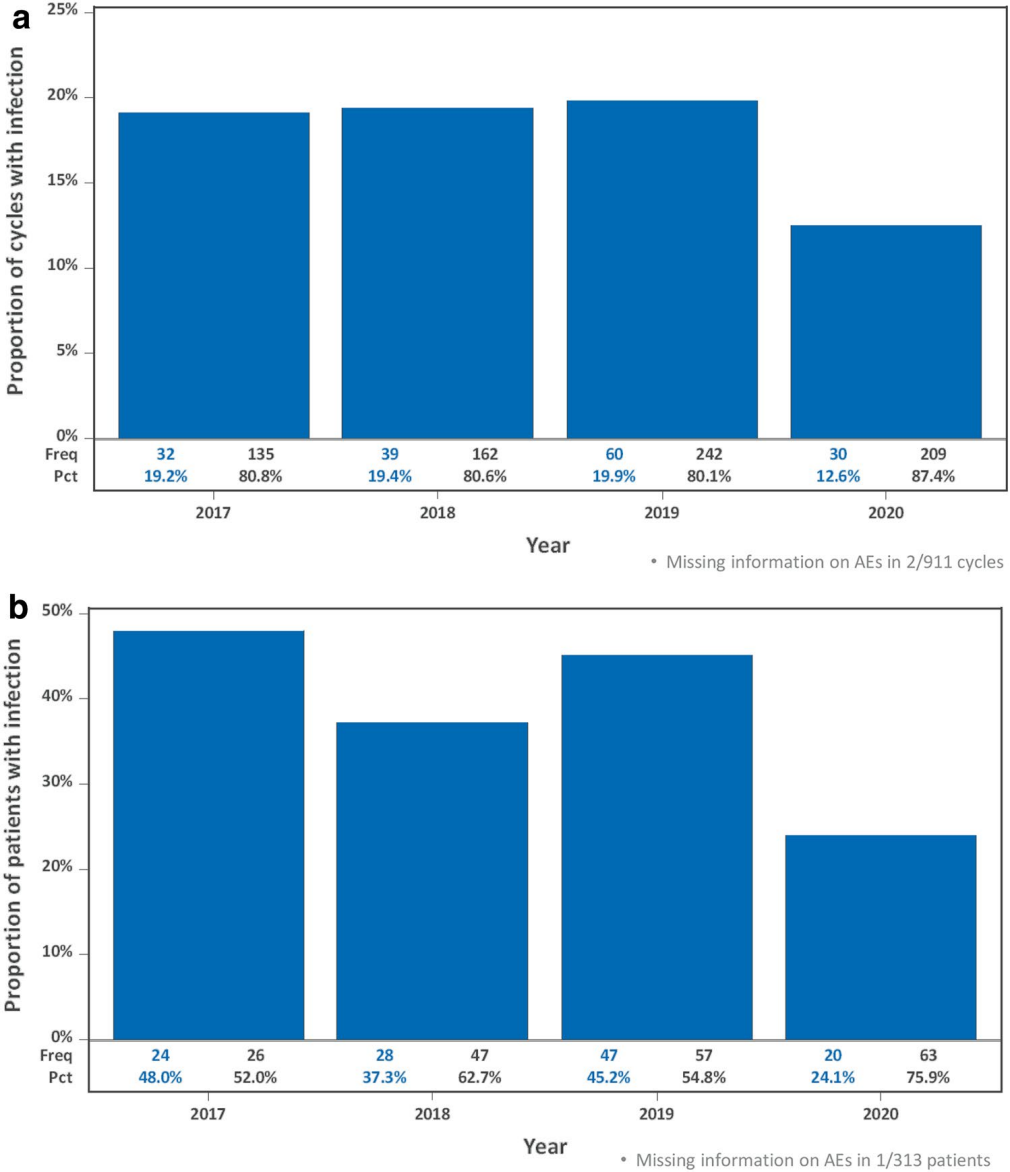
**a** Analyzed cycles (March–June)—Infections per year. Missing information on Adverse Events (AEs) in 2/911 cycles. **b** Analyzed patients (March–June)—Infections per year. Missing information on AEs in 1/313 patients

During the observed period, an infection of any grade was documented in 161 (17.7%) of all 909 analyzed cycles with sufficient information on adverse events (Fig. 2a). Overall, 119 (38.1%) of 312 patients with information on adverse events had an infection during the respective periods (Fig. 2b).

Patients aged 30–39 years had the highest proportion of cycles with an infection with 60 of 276 cycles (21.7%). There was a trend towards lower infection rates with ongoing chemotherapy per patient, with highest rate of infection observed in cycle 1 (40 of 195 cycles; 20.5%).

There was a significantly lower rate of infection during the COVID-19 lockdown period. Infections occurred in 30 (12.6%) of 239 cycles within the COVID-19 lockdown in comparison to a total of 131 (19.6%) of 670 cycles in 2017–2019 [OR 0.574 (95% CI 0.354–0.930), *P* = 0.024 adjusted for age, cycle number and WBC count]. In the reference period, we observed a relatively steady infection rate (range 19.2–19.9% in the years 2017–2019).

Similar results can be seen for the analyzed patients in the respective period per year: 20 (24.1%) of 83 patients had an infection during the COVID-19 lockdown period in comparison to a total of 99 (43.2%) of 229 patients in the respective period of the years 2017–2019 [range 37.3–48.0%; OR 0.405 (95% CI 0.226–0.725), *P* = 0.0023 adjusted for age, number of applied cycles and baseline WBC count; Table 2].

**Table 2:** Logistic regression on infections per cycle

A) Infections per cycle, *N* = 908^a^				
Parameter		Odds ratio	(95% CI)	*P*
Age at enrollment [8]	Cont	0.991	(0.974–1.008)	0.30
Cycle number	Cont	1.001	(0.877–1.142)	0.991
WBC count at start of cycle	Cont	1.021	(0.988–1.055)	0.21
Cycle start in COVID-19 protection period	Yes vs. No	0.574	(0.354–0.930)	0.024

A) Infections per patient, *N* = 312^b^				
Parameter		Odds ratio	(95% CI)	*P*
Age at enrollment [8]	Cont	0.988	(0.968–1.009)	0.26
Number of documented cycles	Cont	1.363	(1.149–1.618)	0.0004
Baseline WBC count	Cont	0.981	(0.940–1.023)	0.37
Treated in COVID-19 protection period	Yes vs. No	0.405	(0.226–0.725)	0.0023

^a^3 of 911 analyzed cycles excluded due to missing information on adverse events *(n* = 2) or WBC count (*n* = 1)

^b^1 of 313 analyzed patients excluded due to missing information on adverse events

Within the documented infectious diseases, the biggest difference between lockdown and the years before can be seen for unspecific infections: there were 39 cycles (5.8%) with an “Infection NOS” during 2017–2019 in comparison to 5 cycles (2.1%) during COVID-19 lockdown. Other common infectious complications under chemotherapy such as febrile neutropenia did not show a major difference: There were 37 cycles with febrile neutropenia (5.5%) in the years 2017–2019 compared to 15 cycles (6.3%) during the COVID-19 lockdown. SAEs have been reported in 56 of all analyzed cycles (6.2%) with similar rates in the lockdown period and the years before (Table 3). Specific infection characteristics can be found in Table 3.

**Table 3:** Infection characteristics per cycle

	Not in COVID-19 lockdown period (*N* = 670) (%)	In COVID-19 lockdown period (*N* = 239) (%)	Total (*N* = 909^a^) (%)
Any infectious disease	131 (19.6)	30 (12.6)	161(17.7)
Infectious disease			
Pneumonia	1 (0.1)	2 (0.8)	3 (0.3)
Stomatitis	1 (0.1)	1 (0.4)	2 (0.2)
Gastroenteritis	1 (0.1)	–	1 (0.1)
Abscess jaw	1 (0.1)	–	1 (0.1)
Sepsis	1 (0.1)	–	1(0.1)
Urinary tract infection	–	1 (0.4)	1 (0.1)
Febrile neutropenia	37 (5.5)	15 (6.3)	52 (5.7)
Etiologic agent			
Bacteria (Escherichia infection, Staphylococcal sepsis, Campylobacter infection, Klebsiella bacteriaemia)	8 (1.2)	–	8 (0.9)
Virus (Herpes/ Herpes zoster)	1 (0.1)	1 (0.4)	2 (0.2)
Virus (Varicella zoster, Coronavirus, Influenza B)	2 (0.3)	1 (0.4)	3 (0.3)
Fungi (Oral candidiasis, Candida sepsis)	2 (0.3)	–	2 (0.2)
Not specified			
Infection NOS	39 (5.8)	5 (2.1)	44 (4.8)
Bacterial infection NOS	19 (2.8)	1 (0.4)	20 (2.2)
Viral infection NOS	11 (1.6)	2 (0.8)	13 (1.4)
Fungal infection NOS	5 (0.7)	–	5 (0.6)
Bacterial and fungal infection NOS	2 (0.3)	1 (0.4)	3 (0.3)
Highest CTCAE grade			
1	34 (5.1)	1 (0.4)	35 (3.9)
2	32 (4.8)	7 (2.9)	39 (4.3)
3	58 (8.7)	18 (7.5)	76 (8.4)
4	7 (1.0)	4 (1.7)	11 (1.2)
Severity			
Serious	41 (6.1)	15 (6.3)	56 (6.2)
Non-serious	90 (13.4)	15 (6.3)	105 (11.6)

Data presented as *n* (%)

*NOS* not otherwise specified, *CTCAE* common terminology criteria for adverse events

^a^Information on adverse events missing in 2 of 911 analyzed cycles

CTCAE grade 3/4 infections occurred in 65 (9.7%) of 670 cycles in 2017–2019 and in 22 (9.2%) of 239 cycles during the COVID-19 protection period, while there was a strong trend towards fewer low-grade infections of CTCAE grade 1/2 during the COVID-19 lockdown period (8 cycles, 3.3%) compared with the 2017–2019 reference period (66 cycles, 9.9%) (Table 3).

We further evaluated the difference in use of supportive measures by period. There was neither a significant difference in hospitalizations during the analyzed cycles [OR 1.048 (95% CI 0.575–1.910), *P* = 0.88 adjusted for age and cycle number] or the rate of dose level reductions [OR 0.901 (95% CI 0.443–1.833), *P* = 0.77 adjusted for age and cycle number] nor the rate of intensified antibiotic prophylaxis [OR 1.138 (95% CI 0.635–2.040), *P* = 0.66 adjusted for age and cycle number]. Detailed information can be found in Table 4.

**Table 4:** Supportive measures per cycle

	Not in COVID-19 lockdown period (*N* = 671)	In COVID-19 lockdown period (*N* = 240)	Total (*N* = 911)
Hospitalization^a^			
Hospitalization during cycle	313/670 (46.7)	113 (47.1%)	426/910 (46.8%)
Number of hospital days per cycle	5 (1–31)	6 (1–20)	5 (1–31)
ICU admission during cycle	1/670 (0.1%)	1 (0.4%)	2/910 (0.2%)
Number of ICU days per cycle	13 NA	4 NA	8.5 (4–13)
Dose level reductions^b^			
Baseline level	30 (4.5%)	12 (5.0%)	42 (4.6%)
Level 1	7 (1.0%)	0	7 (0.8%)
Level 2	30 (4.5%)	9 (3.8%)	39 (4.3%)
Level 3	129 (19.2%)	49 (20.4%)	178 (19.5%)
Full dose	475 (70.8%)	170 (70.8%)	645 (70.8%)
Prophylaxis			
Intensified antibiotic prophylaxis applied	363 (54.1%)	139 (57.9%)	502 (55.1%)
Days with intensified antibiotic prophylaxis per cycle	7 (1–21)	7 (1–21)	7 (1–21)

Data presented as *n* (%), *n*/Total (%) or median (range)

*ICU* intensive care unit, *NA* not applicable

^a^Information on hospitalization missing in 1 of 911 analyzed cycles

^b^eBEACOPP dose levels are defined as follows: full dose: cyclophosphamide 1250 mg/m^2^ on day 1, doxorubicin 35 mg/m^2^ on day 1, etoposide 200 mg/m^2^ on days 1–3, procarbazine 100 mg/m^2^ on days 1–7, prednisone 40 mg/m^2^ on days 1–14, vincristine 1.4 mg/m^2^ (max. 2 mg in total) on day 8, and bleomycin 10 mg/m^2^ on day 8. Level 3: reduce cyclophosphamide to 1100 mg/m^2^ and etoposide to 175 mg/m^2^. Level 2: reduce cyclophosphamide to 950 mg/m^2^ and etoposide to 150 mg/m^2^. Level 1: reduce cyclophosphamide to 800 mg/m^2^ and etoposide to 125 mg/m^2^. Baseline: reduce cyclophosphamide to 650 mg/m^2^, doxorubicin to 25 mg/m^2^ and etoposide to 100 mg/m^2^

## Discussion

The COVID-19 lockdown measures have led to a unique situation, in which we could analyze the impact of prophylactic self-isolation and face masking on the incidence of infections during chemotherapy within a uniformly treated and well-documented cohort of mainly young HL patients.

The most important finding of our analysis is the significant and clinically relevant decrease in the rate of infectious complications during the COVID-19 protection period in comparison to the respective periods in the years 2017–2019. During the COVID-19 lockdown, there was a rate of 12.6% infections in the analyzed cycles in comparison to a rate of approximately 20% in the previous years. This was also true for the rate of infection per patient with 24.1% in 2020 compared to 37.3–48.0% in the years 2017–2019.

This decrease was not equally strong in all subgroups of infections. The strongest effect was evident for infections that were not further classified in the study documentation, i.e. with an unknown type of etiologic agent. We assume that the majority of these is accounted for by environmental pathogens such as viruses causing upper respiratory tract infections, for which the detection rate of any viral pathogen is only up to 36% even in symptomatic patients [17]. This assumption is supported by our results that show a trend towards fewer low CTCAE grade infections during COVID-19 lockdown as compared with the years before.

On the other hand, the rate of febrile neutropenia has stayed relatively stable over the past years and during COVID-19 lockdown. This is in accordance with the assumption that severe neutropenic infections are often caused by pathogens from the patient’s individual microbiome, e.g. *Escherichia coli* infections [18, 19]. Consequently, protection measures such as the ones during COVID-19 lockdown do not have an effect on these more endogenous infections, while exerting a relevant effect on transmitted infections.

Importantly, our findings are in line with data on the activity of several other respiratory viruses in Germany published by the “Clinical Virology Network”, which show that the activity of Influenza A was much lower in 2020 than in 2019. There were only about 430 positive findings for Influenza A in the period of 01 March–30 June 2020, but roughly 4300 positive findings in the respective months of 2019. This is also true for RSV (~ 220 positive findings in 2020 vs. ~ 520 positive findings in 2019) or the Rhinovirus (~ 270 positive findings in 2020 vs. ~ 750 positive findings in 2019) [20]. Similar data were presented by Chan et al. who showed that in Hong Kong, the influenza season was 63.2% shorter, the number of institutional influenza-like-illness (ILI) outbreaks was 68.4% lower and the number of deaths from influenza (laboratory confirmed) in adults was 62.3% lower in the 2019–20 influenza season compared to 2015–19 [21]. Hence, our study and similar data [20, 21] highlight the effectiveness of the COVID-19 protection measures on the rate of acute infections in the general population and of acute infectious complications in HL patients under chemotherapy.

Limitations of our analysis need to be mentioned. First, the relatively low infection rate in 2020 might be a result of reporting bias since we must assume that more recent documentation is missing more often. In order to exclude an effect of fewer cycles being documented per patient, we primarily analyzed the infection rate among documented cycles instead of among patients. Bias might then still occur if documentation of cycles with infection would be missing or delayed more likely than that of cycles without infection. We thus checked the infection rate among cycles starting between May and August 2019 as documented in the dataset from statistical monitoring in 09/2019 vs. 11/2020. In 09/2019 the infection rate was 21% on the basis of 168 documented cycles, while in 11/2020 it was 20% among 290 documented cycles. Reporting bias is thus unlikely to influence our results. Second, we recognize that our study cohort consists of mainly young HL patients and observed effects might not be fully applicable to older patients or other cancer patients treated with different regimens. However, the data on the effect of the COVID-19 lockdown measures on the general population included patients from all age groups [15]. Since these data are in line with our results, we assume that the same effect could be observed in older patients or other malignancies as well. Third, due to the retrospective nature of our analysis, we are not able to definitively assess whether the observed effect can be primarily attributed to the patients’ self-isolation and face masking or the general decrease of air-borne infections during the lock-down period. While our study can thus not prove a causal relationship, we analyzed the confounding factors thoroughly and conclude that a causal relationship is at least highly probable.

Strengths of our analysis include the large, well-documented, uniformly treated study population. The reporting trial sites included both university hospitals and small practices throughout Germany and therefore mirror the real-world setting. The documentation of infectious complications was thorough so that detailed information could be derived and analyzed. Accordingly, the observed effects seem to be robust and reliable.

In conclusion, we recommend to inform cancer patients on the significant protective effect of prophylactic social distancing and face covering on the occurrence of communicable infections. The reliable evidence derived from our observation may support patients in their decision on whether or not they adopt these protective measures into their life while they are treated with chemotherapy for cancer.

## Supplementary Information

Below is the link to the electronic supplementary material.

Supplementary file1 (PDF 155 KB)
