# Supplementary material for: Impact of the first COVID-19 lockdown in Germany on the rate of acute infections during intensive chemotherapy for Hodgkin lymphoma
Source: Infection. 2022 Feb 19;50(4):925–32. doi: 10.1007/s15010-022-01765-3 (PMC8857743; doi:10.1007/s15010-022-01765-3)
Supplement: Supplementary file 1 — Supplementary file1 (PDF 155 KB) [file 15010_2022_1765_MOESM1_ESM.pdf]

Appendix

Flow Chart saved as seperate file

Supplementary Figure 1. Study design. In the main study, patients are randomized to receive chemotherapy with escalated BEACOPP (dose-escalated bleomycin, etoposide, doxorubicin, cyclophosphamide, vincristine, procarbazine and prednisolone) (standard group) or with BrECADD (brentuximab vedotin, etoposide, cyclophosphamide, doxorubicin, dacarbazine and dexamethasone) (experimental group). After the first two cycles, a restaging is performed by contrast-enhanced computed tomography (ceCT) and positron-emission tomography (FDG PET/CT) in all patients in order to guide response-adapted continuation of therapy consisting of 4 or only 2 additional cycles of randomized chemotherapy in case of a PET positive or negative staging result, respectively. A second restaging will be performed after completion of chemotherapy; Patients with PET-positive residual disease will receive local irradiation, while patients in complete remission do not receive radiotherapy.

Supplementary List 1. List of predefined MeDDRA codes for SAEs included in the analysis

<u>System Organ Class (SOC)</u>

- Infections and infestations

<u>High Level Group Term (HLGT)</u>

- Infections and infestations congenital
- Ocular infections, irritations and inflammations
- Gastrointestinal infections
- Central nervous system infections and inflammations
- Female reproductive tract infections and inflammations
- Male reproductive tract infections and inflammations
- Respiratory tract infections
- Skin and subcutaneous tissue infections and infestations
- Vascular inflammations
- Microbiology and serology investigations

<u>High Level Term (HLT)</u>

- Cardiac infections and inflammations NEC
- Inner ear infections and inflammations

- Salivary gland infections and inflammations
- Dental and periodontal infections and inflammations
- Gingival infections
- Gastrointestinal inflammatory disorders NEC
- Rectal inflammations NEC
- Oral soft tissue infections, Diverticulum inflammations
- Peritoneal and retroperitoneal disorders
- Bile duct infections and inflammations
- Cholecystitis and cholelithiasis
- Hepatic infections (excl viral), Hepatic viral infections, Hepatocellular damage and hepatitis NEC
- Hepatic and hepatobiliary disorders NEC
- Bursal disorders
- Synovial disorders
- Infectious arthritis
- Musculoskeletal and connective tissue infections and inflammations NEC
- Soft tissue disorders NEC
- Bone and joint infections (excl arthritis)
- Muscle infections and inflammations
- Bladder infections and inflammations
- Genitourinary tract infections and inflammations NEC
- Breast infections and inflammations
- Pleural infections and inflammations

Preferred Term (PT)

- Febrile neutropenia
- Stomatitis
